# Oral Immunotherapy for Pollen Allergy Using T-Cell Epitope-Containing Egg White Derived from Genetically Manipulated Chickens

**DOI:** 10.1371/journal.pone.0048512

**Published:** 2012-10-29

**Authors:** Yoshinori Kawabe, Yuuki Hayashida, Kensaku Numata, Shota Harada, Yoshifumi Hayashida, Akira Ito, Masamichi Kamihira

**Affiliations:** Department of Chemical Engineering, Faculty of Engineering, Kyushu University, Fukuoka, Japan; University of Manitoba, Canada

## Abstract

Peptide immunotherapy using T-cell epitopes is expected to be an effective treatment for allergic diseases such as Japanese cedar (*Cryptomeria japonica*; Cj) pollinosis. To develop a treatment for pollen allergy by inducing oral tolerance, we generated genetically manipulated (GM) chickens by retroviral gene transduction, to produce a fusion protein of chicken egg white lysozyme and a peptide derived from seven dominant human T-cell epitopes of Japanese cedar pollen allergens (cLys-7crp). The transgene sequence was detected in all chickens transduced with the retroviral vector. Transduction efficiency in blood cells correlated to transgene expression. Western blot analysis revealed that cLys-7crp was expressed in the egg white of GM hens. Mice induced to develop allergic rhinitis by Cj pollinosis were fed with cLys-7crp-containing egg white produced by GM chickens. Total and Cj allergen (Cry j 1)-specific IgE levels were significantly decreased in allergic mice fed with cLys-7crp-containing egg white compared with allergic mice fed with normal egg white. These results suggest that oral administration of T-cell epitope-containing egg white derived from GM chickens is effective for the induction of immune tolerance as an allergy therapy.

## Introduction

Japanese cedar (*Cryptomeria japonica*; Cj) pollinosis is a serious type I allergic disease in Japan [1]. More than 25% of Japanese citizens suffer from Cj pollinosis and the number of patients has constantly increased over the years [2]. Type I allergic diseases, including Cj pollinosis, are characterized by the elevation of immunoglobulin E (IgE) levels and mast cell degranulation, followed by the release of histamine and other chemical mediators of allergy [3]. The main strategy for treating allergic diseases is based on pharmacological therapy, where inflammatory responses mediated by chemical mediators such as histamine and leukotriene, are inhibited using anti-inflammatory agents. A periodic recurrence of pollinosis is observed in patients since the therapeutic approach represents a symptomatic treatment only.

Hyposensitization therapy has been used as a conventional allergen-specific immunotherapy [4] to try and cure patients completely. A stepwise subcutaneous administration of an escalating dose of the specific crude allergen extracts has been used for the treatment for allergies. However, this therapy has some disadvantages, such as the requirement of long-term administration of allergens under the control of physicians and the possibility of triggering an adverse reaction such as acute anaphylaxis by allergen administration [5], [6]. Thus, it is highly desirable to establish a more convenient and curative immunotherapy.

Immunotherapy using allergen-specific T-cell epitopes is thought to be a safe and effective treatment for the control of IgE-mediated allergic diseases [7]. In Cj pollinosis, two cedar pollen proteins, Cry j 1 (40–45 kDa) and Cry j 2 (37 kDa), were identified as major pollen allergens [8]. After the complementary DNA (cDNA) clones encoding Cry j 1 [9] and Cry j 2 [10] were isolated and sequenced, the dominant human T-cell epitopes were identified from proliferative responses of peripheral blood mononuclear cells from many patients with Cj pollinosis [11], [12]. Among them, seven major human T-cell epitopes derived from Cry j 1 and Cry j 2 were selected to construct an artificial protein (7crp) used for vaccination. Takaiwa and co-workers reported an oral immunotherapy regime using the T-cell epitope peptides and 7crp, using a transgenic rice-based allergy vaccine [13], [14]. Oral administration of allergens induces a state of systemic unresponsiveness against the administered allergens, although the mechanisms underlying oral tolerance are still not well understood [15]. Oral administration of transgenic rice containing the major mouse T-cell epitopes derived from Cry j 1 and Cry j 2 [13] or 7crp [14] resulted in inhibition of the allergy-associated Th2 reactions such as decreased total and allergen-specific IgE levels, change in cytokine production profiles, and a reduction in sneezing. Although this strategy used only T-cell epitope peptides, fusion proteins containing T-cell epitopes have also been used as immunogens for effective hyposensitization therapy of allergy. For example, T-cell epitopes fused with an invariant chain to promote the processing of epitope peptides into the MHC class II loading pathway [16], [17], and an allergen protein fused with an Fc region derived from immunoglobulin [18] for improved stability in the bloodstream, were used for vaccination. In order to produce such complicated artificial proteins containing T-cell epitopes to induce a more effective oral tolerance, the selection of host cells may be important in terms of proper protein folding and post-translation modifications such as glycosylation [19].

Transgenic avian species such as chicken and quail have attracted much attention as “living bioreactors” for the next generation platform of biopharmaceutical protein production. Compared with other mammalian bioreactors such as the goat and cow, chickens have several advantages, such as rapid sexual maturity, small space requirement for breeding, high protein yield of eggs, and a similarity of *N*-linked oligosaccharide structures to humans [20], [21]. We previously generated genetically manipulated (GM) chickens and quails using retroviral vectors for gene transfer and succeeded in producing recombinant proteins such as antibodies [22]–[24] and human erythropoietin [25], [26] in eggs and serum of GM avian species.

This study aims to investigate whether oral tolerance against Cj pollinosis can be induced using egg white containing T-cell epitopes produced by GM chickens. First, GM chickens producing 7crp fused with chicken egg white lysozyme (cLys-7crp) were generated using a retroviral vector. Then, the cLys-7crp-containing egg white was orally administered to Cj pollinosis model mice to investigate its therapeutic effects. The present study demonstrates that peptide immunotherapy through oral administration of epitope peptide-containing egg white produced by GM chickens is a promising approach for allergy therapy.

## Materials and Methods

### Animals

Laid, fertilized chicken eggs were obtained from a local breeder in Fukuoka (Farmhouse, Fukuoka, Japan). Chickens were housed in individual cages on a 16-h light/8-h dark cycle, at a controlled temperature with water and food available *ad libitum*. Mice were obtained from Japan SLC (Shizuoka, Japan) and bred in a temperature- and light-controlled environment. All animal experiments in this study were approved by the ethics committee for animal experiments of the Faculty of Engineering, Kyushu University (A19-116-1, A22-245-1 and A23-145-1).

### Retroviral vector construction

**Figure 1:**
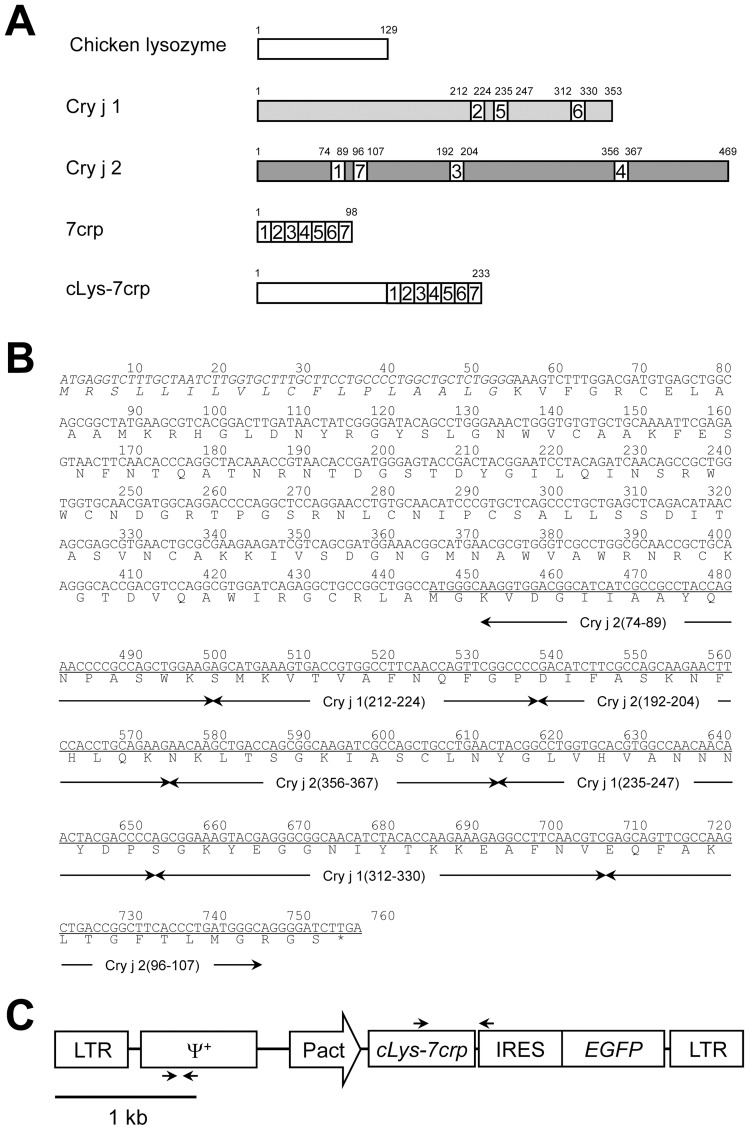
Design of cLys-7crp to produce T-cell epitopes derived from Japanese cedar pollen allergens. (**A**) Schematic representations of chicken egg white lysozyme, Cry j 1, Cry j 2, 7crp and cLys-7crp fusion proteins. The numbers of amino acids are shown above the respective proteins. Seven major human T-cell epitopes recognized by many Cj pollinosis patients were selected from Cry j 1 and Cry j 2 proteins and a hybrid epitope peptide (7crp) was designed by accumulating the selected seven major human T-cell epitopes. 7crp protein was fused with the C-terminal of chicken lysozyme (cLys-7crp). (**B**) Nucleotide and amino acid sequences of cLys-7crp. The position of each T-cell determinant is indicated below the amino acid sequence. The secreted signal region of chicken lysozyme is indicated in italics. The 7crp region is underlined. The termination codon (TGA) is marked with an asterisk. (**C**) Structure of the retroviral vector for the production of cLys-7crp. LTR, long terminal repeat derived from mouse stem cell virus (MSCV); Ψ+, virus packaging signal sequence derived from MSCV; Pact, chicken β-actin promoter; *cLys-7crp*, cLys-7crp gene; IRES, internal ribosomal entry site sequence from encephalomyocarditis virus (EMCV); *EGFP*, enhanced green fluorescent protein gene. The annealing sites of RT-PCR and genomic PCR primers are indicated as arrows above and below the vector structure, respectively.

A schematic representation of the retroviral vector used in this study is shown in Fig. 1. The retroviral vector plasmid (pQMSCV), designed for the production of retroviral vectors based on a mouse stem cell virus (MSCV), was described previously [27]. A chicken egg white lysozyme (cLys) gene was amplified by PCR from chicken oviduct cDNA, using the primers, 5′-CCG CTC GAG AAC ATG AGG TCT TTG CTA ATC TTG G-3′ and 5′-ATT CCA TGG CCA GCC GGC AGC CTC TG-3′, to append *Xho*I and *Nco*I digestion sites (underlined) onto either end of the PCR product. The PCR was initiated using *Thermococcus kodakaraensis* (KOD) plus polymerase (Toyobo, Osaka, Japan) at 94°C for 2 min, followed by 30 cycles of amplification at 94°C for 15 s, 57°C for 30 s and 68°C for 45 s. The PCR product was digested with the respective restriction enzymes and ligated into *Xho*I- and *Nco*I-digested pETBlue-2 (Novagen, Merck KGaA, Darmstadt, Germany) to generate pET/cLys. A double stranded DNA oligonucleotide corresponding to the sequence of 7crp, an artificial protein comprising of seven human T-cell epitopes derived from Cry j 1 (212–224, 235–247, 312–330) and Cry j 2 (74-89, 96–107, 192–204, 356–367) was chemically synthesized according to the chicken codon usage and introduced into the pBluescript KS (−) (Toyobo) to generate pBlue/7crp. The 7crp gene was amplified by PCR from pBlue/7crp using the following primers, 5′-ACG CCC ATG GCA AGG TGG ACG GCA T-3′ and 5′-CGG GCT GCA GGA ATT CG-3′ to append *Nco*I and *Eco*RI digestion sites (underlined) onto either end of the PCR product. The PCR was initiated using KOD plus DNA polymerase (Toyobo) at 94°C for 2 min, followed by 30 cycles of amplification at 94°C for 15 s, 57°C for 30 s and 68°C for 25 s. The PCR product was digested with the respective restriction enzymes and ligated into *Nco*I- and *Eco*RI-digested pET28a (Novagen) to generate pET28a/7crp. The DNA fragment encoding the cLys gene prepared from pET/cLys was ligated into pQMSCV/CMVHBD3/IRES/EGFP [28] together with the DNA fragment encoding the 7crp gene prepared from pET28a/7crp to generate pQMSCV/cLys-7crp/IRES/EGFP. The constitutive chicken β-actin promoter (ΔA) prepared from pMSCV/GΔAscFv-Fc [22] was ligated into pQMSCV/cLys-7crp/IRES/EGFP to generate pQMSCV/ΔAcLys-7crp/IRES/EGFP (7659 bp), in which the cLys-7crp gene was expressed under the control of chicken β-actin promoter and the enhanced green fluorescence protein (EGFP) gene was bicistronically expressed using an internal ribosomal entry site (IRES) derived from the encephalomyocarditis virus (EMCV). All DNA sequences derived from PCR products on the plasmid vectors were confirmed by a Prism 3130 Genetic Analyzer (Applied Biosystems, Foster City, CA, USA).

### Retroviral vector production and microinjection into chicken embryos

Vesicular stomatitis virus (VSV)-G pseudotyped pantropic retroviral vector was produced as reported previously [27]. The viral titer was determined from the population of EGFP-expressing cells in NIH3T3 cells infected with the viral solution by flow cytometry (BD FACSCalibur™, Franklin Lakes, NJ, USA). Microinjection of the viral solution and embryo cultures were performed as reported previously [22]. The concentrated viral solution was adjusted to a titer between 8.3×10^7^–2.6×10^9^ infectious units (IU)/ml and injected into the heart of chicken embryos after 55 h incubation (stage 13–16 according to Hamburger and Hamilton [29]). The injection volume was 2.0–4.0 μl per embryo.

### Determination of transgene copy number by real-time PCR

Genomic DNA was extracted from blood cells and tissues (brain, heart, skeletal muscle, liver, spleen, kidney, oviduct and ovary) of GM chickens using a genomic DNA preparation kit (Mag extractor genome; Toyobo). Real-time PCR (Light Cycler 1.5 Instrument; Roche Diagnostics GmbH, Mannheim, Germany) was used to assess the copy number of transgenes in the cells using 20 ng of genomic DNA as a template. PCR was performed using hybridization probes and the quantitative PCR reagent (Light Cycler FastStart DNA Master HybProbe; Roche) according to the manufacturer's instructions except for the addition of 4 mM MgCl_2_, under the following conditions: 95°C for 10 min, followed by 40 cycles of 95°C for 10 s, 58°C for 15 s, and 72°C for 5 s and then 40°C for 30 s. The primers used to amplify the retroviral vector sequence were 5′-CAA GAA GAG ACG TTG GGT TAC-3′ and 5′-CTC CCA GGT CAC GAT GT-3′ for the MSCV packaging region (173 bp). The hybridization probe sequences were 5′-GGC CAG GTG AAA AGA CCT TGA TCT TAA CCT-3′ labeled with fluorescein isothiocyanate at the 3′ end and 5′-GGT GAT GAG GTC TCG GTT AAA GGT GCC-3′ labeled with LCRed640 at the 5′ end for the MSCV packaging signal region. The primers and hybridization probes were synthesized by Hokkaido System Science (Sapporo, Japan) and Nihon Gene Research Laboratories (Sendai, Japan), respectively. Calibration curves for the real-time PCR were created using a dilution series of pQMSCV/ΔAcLys-7crp/IRES/EGFP as the standard template.

### Preparation of anti-7crp antisera

A DNA fragment encoding a 7crp gene including His-tag prepared from pET28a/7crp was subcloned into the pGEX-6P-2 plasmid (GE Healthcare, Amersham, UK) to generate pGEX/7crp. Competent *Escherichia coli* cells (Rosetta2 [DE3] stain; Novagen) transformed with pGEX/7crp was grown in LB medium at 37°C to an optical density absorbance at 600 nm of 0.4–0.6 and then induced with 0.5 mM isopropyl-β-D-thiogalactopyranoside for 4 h at 37°C. The *E. coli* cells were collected by centrifugation. After washing the cells with washing buffer (10 mM Tris-HCl [pH 7.8], 1 mM EDTA, 0.1 M NaCl), cells were resuspended in phosphate-buffered saline (PBS) containing 1 mM dithiothreitol (Wako Pure Chemical Industries, Osaka, Japan), 100 μg/ml phenylmethylsulfonyl fluoride (PMSF; Sigma-Aldrich, St. Louis, MO, USA) and 2 mM imidazole (Wako Pure Chemical Industries), and disrupted by sonication (Branson Ultrasonics, Danbury, CT, USA). The sonicated solution was centrifuged at 12,000× *g* for 15 min at 4°C, and the insoluble matter containing the 7crp inclusion body was dissolved in 8 M Urea. After filtering using a 0.45 μm cellulose acetate filter (Advantec, Tokyo, Japan), the His-tagged 7crp protein was purified using a His Trap crude Kit (GE Healthcare) according to the manufacturer's instructions except for the addition of 8 M urea to the binding buffer and elution buffer. The purified protein was analyzed by electrophoresis on a 10% sodium dodecyl sulfate (SDS)-polyacrylamide gel and visualized by SimplyBlue™ SafeStain (Invitrogen, Carlsbad, CA, USA).

To prepare the anti-7crp antisera, the purified protein was diluted with dilution buffer (50 mM Tris-HCl [pH 7.4], 150 mM NaCl, 0.5 M Arginine) to a final concentration of 20 μg/ml. The 7crp solution (250 μl) was mixed with 250 μl of complete Freund's adjuvant (DIFCO, Detroit, MI, USA), and the mixture was injected intraperitoneally into six BALB/c female mice (Japan SLC) for the first immunization. After 14 days, a booster immunization was performed using the mixture of 7crp solution with incomplete Freund's adjuvant (DIFCO). The injection was repeated again after two weeks. Two days after the final booster, blood was collected from the heart of immunized mice, and antisera prepared using the standard procedure. Antisera were used for western blot analysis to detect 7crp proteins, as described below.

### Transgene expression analysis

SDS-polyacrylamide gel electrophoresis (SDS-PAGE) was followed by western blot analysis to detect cLys-7crp. The cell lysates of NIH3T3 cells transduced by the MSCV/ΔAcLys-7crp/IRES/EGFP retroviral vector and chicken lysozyme protein (Wako Pure Chemical Industries) were used as positive controls, and normal NIH3T3 cells and wild-type chicken egg white were used as negative controls. Egg white samples (2 μl) were boiled in the SDS-PAGE sample buffer containing 2-mercaptoethanol, separated in a 12% (w/v) SDS-polyacrylamide gel and transferred onto polyvinylidene fluoride membranes (GE Healthcare). After blocking with Tris buffered saline-Tween 20 (25 mM Tris-HCl [pH 7.4], 150 mM NaCl and 0.05% (v/v) Tween 20) containing 5% (w/v) non-fat milk at 4°C, 7crp proteins on the membranes were detected using mouse anti-7crp antisera at 1∶2,000 dilution and a peroxidase (POD)-conjugated goat anti-mouse IgG secondary antibody (Santa Cruz Biotechnology, Santa Cruz, CA, USA) at 1∶10,000 dilution. To enhance signal detection, Can Get Signal™ solution (Toyobo) was used to dilute the antibody. POD activity was detected using an enhanced chemiluminescence kit (ECL Plus Western Blotting Detection System; GE Healthcare).

For *in situ* GFP visualization, a blue light-emitting diode light (cellular blue LED light, LEDB-3WOF; OptoCode, Tokyo, Japan) was shed on the GM chicken through a GFP filter. The population of GFP-positive blood cells prepared from GM chickens was measured by flow cytometry (BD FACSCalibur™).

Total RNA was extracted from the tissues (brain, heart, skeletal muscle, liver, lung, spleen, kidney, oviduct and ovary) of a GM chicken using a commercially available kit (RNAiso plus; Takara Bio, Otsu, Japan) according to the manufacturer's protocol. Total RNAs were treated with DNase I (Wako Pure Chemical Industries) and then converted to cDNA using oligo-dT primer and ReverTra Ace reverse transcriptase (Toyobo). The cDNAs from each sample were subjected to PCR. The following primers were used; 5′-ACC GTA ACA CCG ATG GGA GTA-3′ and 5′-ACA CCG GCC TTA TTC CAA-3′ for the cLys-7crp sequence and 5′-CAC AGA AGA CGG TGG ATG GC-3′ and 5′-GAG ACA TTG GGG GTT GGC A-3′ for the glyceraldehyde 3-phosphate dehydrogenase (GAPDH) gene. PCR was initiated with DNA polymerase (G-Taq; Cosmo Genetech, Seoul, Korea) at 95°C for 2 min, followed by 35 cycles of amplification at 95°C for 20 s, 56°C for 40 s, 72°C for 10 s, and 72°C for 5 min for final extension.

### Therapeutic protocol for oral administration of egg white containing cLys-7crp to treat Cj pollinosis

For therapeutic trials, we first generated an allergic rhinitis mouse model specific for Cj pollinosis. Mice (B10.S strain; 8 weeks old, female) were sensitized with 10 μg of cedar pollen extract (Cedar Pollen Extract-Cj, cat no. LG-5280; Cosmo Bio, Tokyo, Japan) mixed with 4 mg of aluminum hydroxide gel (Imject Alum; Pierce, Rockford, IL, USA) by subcutaneous injection once a week for three consecutive weeks. Nine weeks after the final sensitization and prior to the intranasal challenge, 10 ng of histamine solution (2 μg/ml histamine dihydrochloride in PBS; Nacalai Tesque Inc., Kyoto, Japan) was administered into both nostrils. Histamine pretreatment was performed to trigger the enhanced nasal responses, as described previously [30]. After the histamine treatment, 5 μl of cedar pollen extract (400 μg/ml in PBS) was dropped into each nostril, using a micropipette, for 5 consecutive days. Five weeks after the intranasal challenge, egg white (0.3 ml containing approximately 0.3 μg cLys-7crp) from a GM chicken (#23) (seven mice) or a non-GM hen (four mice) was orally administered to mice with Cj pollinosis, 5 days a week for four consecutive weeks using a feeding needle (Natsume Seisakusho, Tokyo, Japan). Four weeks after the last feeding, mice were re-immunized intranasally with 2 μg cedar pollen extract in PBS (5 μl per nostril). The oral administration followed by intranasal challenge was defined as one term of therapy, and the therapeutic trial term was repeated again. After the final intranasal challenge for each therapeutic trial term, the number of sneezes accompanied by trembling was measured for 5 min using video recordings.

### Measurement of serum IgE

Sera were prepared from blood obtained from the tail vein of mice two weeks after the intranasal antigen challenge for each therapeutic trial term, at which time the expression of antigen-specific IgE levels peak [31]. Total and Cry j 1-specific IgE in the serum was measured using an enzyme-linked immunosorbent assay (ELISA) and alphaLISA (PerkinElmer, Waltham, MA, USA), respectively. The samples were assayed in triplicate, and IgE amounts were expressed as values relative to average values calculated from naïve mice as unprimed controls, with standard deviations.

For the total IgE assay, 384-well ELISA microtiter plates (cat. no. 3701; Corning, New York, NY, USA) were coated with 30 μl of goat anti-mouse IgE antibody (10 μg/ml, SouthernBiotech, Birmingham, AL, USA) overnight at 4°C. After removal of the coating solutions, the plates were washed with PBS containing 0.05% (v/v) Tween 20 and blocked with 50 μl of 1% (w/v) bovine serum albumin (BSA) in PBS for 1 h at room temperature. After blocking and washing, 30 μl of serum samples diluted at 1∶50 were added to triplicate wells, followed by incubation for 1 h at room temperature. The plates were washed and then 50 μl of goat anti-mouse IgE conjugated with peroxidase (SouthernBiotech) at 1∶4,000 dilution was added to each well. After incubation for 1 h at room temperature, 50 μl of QuantaBlu™ fluorogenic peroxidase substrate (Pierce) was added to the wells. After 30 min, the enzymatic reaction was stopped by adding 50 μl of stop solution according to the manufacturer's protocol. The absorbance of each well was measured at 325/420 nm (Excitation/Emission) using a 2300 EnSpire™ Multilabel Reader (PerkinElmer).

For the Cry j 1-specific IgE assay, alphaLISA was performed according to the manufacturer's protocol. Briefly, goat anti-mouse IgE antibody (100 μg) (SouthernBiotech) was labeled with alphaScreen streptavidin acceptor beads (1 mg) using sodium cyanoborohydride (Sigma-Aldrich) and O-(carboxymethyl) hydroxylamine hemihydrochloride (Sigma-Aldrich). The serum samples were diluted at 1∶3, 25 μg/ml Cry j 1 conjugated with biotin (cat. no. HBL-BC-1, Wako Pure Chemicals) and anti-mouse IgE antibody conjugated with alphaScreen streptavidin acceptor beads were added into triplicate wells of a 384-well microplate (AlphaPlate™-384) for 1 h at 23°C. Thereafter, streptavidin-coated alphaScreen donor beads were added and incubated for 30 min at 23°C. The absorbance of each well was measured at 680/615 nm (Excitation/Emission) using a 2390 EnSpire™ Multilabel Reader (PerkinElmer).

Statistical significance was assessed using an unpaired Student's *t*-test. Values of *p*<0.05 were considered statistically significant.

### Histological analyses

After the final therapeutic trial term, the intranasal challenge using the cedar pollen extract was performed to elicit inflammatory responses by the allergens in the treated mice. Twenty-four hours after the intranasal challenge, lungs were harvested from the mice. For histological evaluation, the lung tissues were washed three times with PBS, fixed in 4% (v/v) paraformaldehyde in PBS and embedded in paraffin. Thin sections (3 μm) were cut and stained with hematoxylin and eosin (H&E) or periodic acid-Schiff (PAS)/hematoxylin. The stained sections were observed under a BZ-9000 microscope (Keyence, Osaka, Japan).

## Results

### Generation of GM chickens producing cLys-7crp

A 7crp gene was chemically synthesized using codons optimized based on the four most abundant egg white protein genes (ovalbumin, lysozyme, ovomucoid, and ovotransferrin). In the preliminary experiment, we constructed a retroviral vector plasmid (pQMSCV/GΔA7crp), in which an expression unit of the 7crp gene including a cLys-derived secretion signal sequence under the control of chicken β-actin promoter was encoded. However, a viral solution with a titer high enough for efficient delivery into embryonic cells could not be prepared using the plasmid. We assumed that the artificial protein comprised of the seven epitopes might have a cytotoxic effect on the viral producer cells. Thus, to reduce the cytotoxic effect, 7crp was produced as a fusion protein with cLys, a major component of egg white proteins. We constructed a retroviral vector plasmid, pQMSCV/ΔALys-7crp/IRES/EGFP, encoding a bicistronic expression cassette of cLys-7crp and EGFP mediated by IRES under the control of chicken β-actin promoter. By changing the production format, the titer of retroviral vector increased to ≥10^8^ IU/ml after ultracentrifugation. The concentrated viral solution was injected into the heart of developing chicken embryos after 55 h incubation [22]. A total of 74 chicken embryos in four experiments were injected with the retroviral solution (2.0–4.0 μl). After the viral injection, the chicken embryos were cultured to hatch. The hatchability of the embryos was in the range of 20–44%, and 31% on average (Table 1). The hatched birds grew normally to maturity and female chickens produced eggs after sexual maturation.

**Table 1:** Retroviral vector injection into chicken embryos and hatchability of virus-injected embryos.

Experiment number	Viral titer (IU/ml)	Number of embryos	
		Injected	Hatched (Hatchability)
1	8.3×10^7^	9	4 (44%)
2	3.8×10^8^	16	7 (44%)
3	1.9×10^8^	15	4 (27%)
4	2.6×10^9^	25	5 (20%)
Total	–	65	20 (31%)

**Figure 2:**
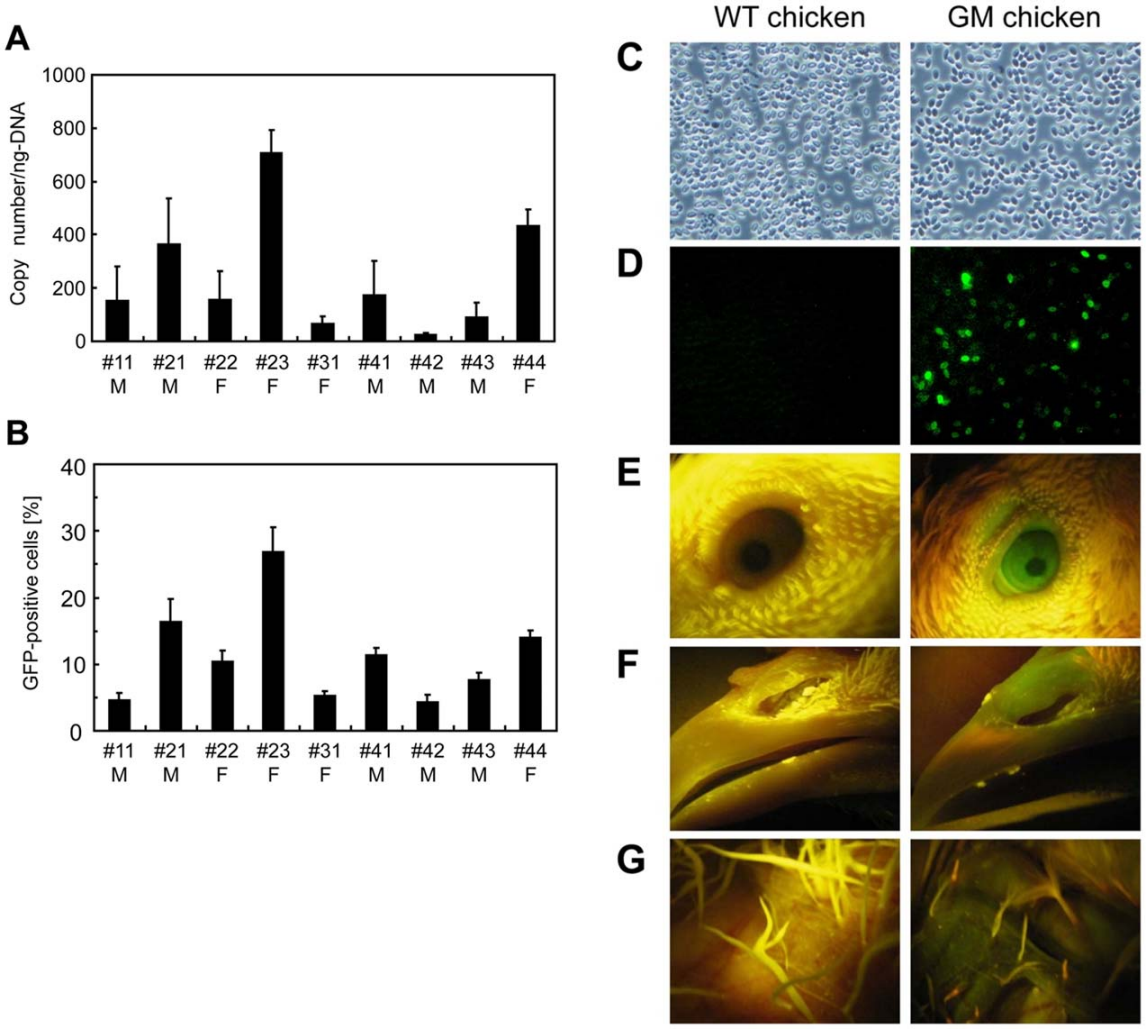
Transgene analysis of GM chickens. (**A**) Mean copy number of the transgene. Quantitative real-time PCR analysis was performed to determine the mean copy number of transgenes in blood cells from GM chickens. Samples were prepared and assayed in triplicate. M, male; F, female. (**B**) Percentage of GFP-positive cells in the blood of GM chickens. M, male; F, female. (**C, D**) Observation of blood cells (**C**, phase contract; **D**, green fluorescence image). Left panels, wild-type chicken; right panels, GM chicken. (**E–G**) Detection of EGFP expression (E, eye; F, beak; G, skin).

Retroviral sequences were detected by PCR using genomic DNA extracted from the chicken blood cells as a template and specific primers for the retroviral packaging signal region. The PCR analysis revealed that all chickens were transduced with the retroviral vector containing the transgene. Transgene transduction was also detected in various tissues and organs such as the brain, heart, liver, lung, spleen, kidney, muscle, oviduct and gonads (data not shown). To further examine the transgene copy number in the blood cells of GM chickens, genomic DNA was subjected to real-time PCR. The copy number varied among the GM chickens, with a mean value of approximately 230 copies per ng of DNA (Fig. 2A) corresponding to 0.6 copies per cell.

It was reported that an EMCV-derived IRES could be utilized for the co-expression of the transgene in chickens [24], [32]. In this study, cLys-7crp and EGFP genes were co-expressed bicistronically mediated by IRES under the control of the ubiquitous chicken β-actin promoter. Therefore, transgene expression was visually detectable by fluorescence microscopy based on the EGFP expression in the blood cells of GM chickens (Fig. 2B, 2D). Chicken red blood cells are nucleated, and EGFP-positive cells were observed in both red and white blood cells. The population and expression level of EGFP-positive cells were higher for white blood cells than those for red blood cells. From the FACS analysis, 4–27% of blood cells were EGFP-positive in the GM chickens (Fig. 2B). The frequency of EGFP-positive cells correlated to the transgene copy number of the GM chickens (Fig. 2A), although the values were not consistent each other. Moreover, EGFP expression was detected throughout the GM chicken body such as the eye (Fig. 2E), beak (Fig. 2F) and skin (Fig. 2G).

**Figure 3:**
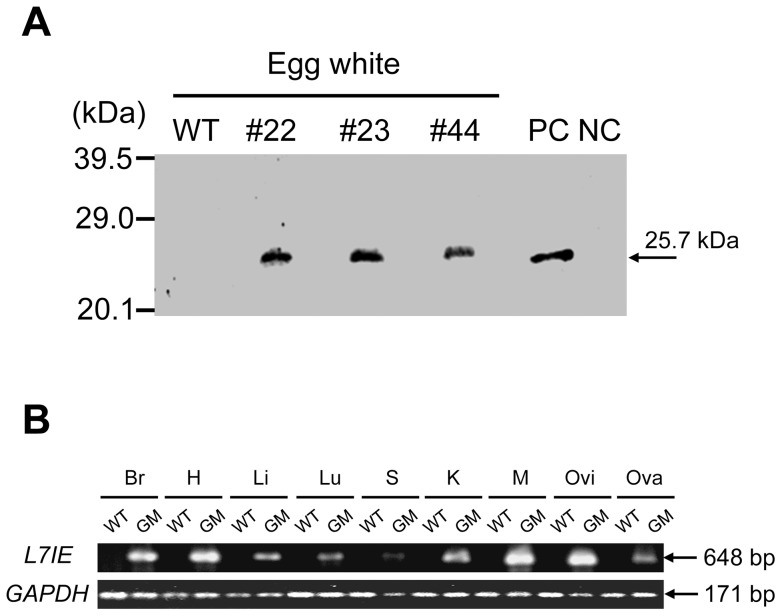
Expression analysis of cLys-7crp. (**A**) Western blot analysis for cLys-7crp in the egg white of GM chickens (#22, #23 and #44). WT, wild-type chicken. 3T3 cells transduced with MSCV/ΔAcLys-7crp/IRES/EGFP retroviral vector and parental NIH3T3 cells were used as a positive control (PC) and negative control (NC), respectively. (**B**) RT-PCR analysis for transgene expression in various tissues (Br, brain; H, heart; Li, liver; Lu, lung; S, spleen; K, kidney; M, muscle; Ovi, oviduct; Ova, ovary) from adult hens. WT, wild-type chicken; GM, GM chicken.

In addition, cLys-7crp expression in the eggs laid by GM hens was detected by western blot analysis using anti-7crp serum (Fig. 3A). According to the band density of western blot analysis, cLys-7crp was produced in the egg white from GM chickens with a concentration of 1.0–1.6 μg/ml-egg white. The cLys-7crp expression in the yolk was negligible (data not shown). However, cLys-7crp expression was not detected in the serum, tissues and organs of GM chickens despite the transgene being expressed under the control of the ubiquitous β-actin promoter (data not shown). RT-PCR analysis was performed to examine the expression of cLys-7crp transcripts in various tissues from a GM chicken, and the transcript was detected in all the tissues tested (Fig. 3B).

### Oral administration of cLys-7crp-containing egg white induced mucosal tolerance to the Cj allergen

**Figure 4:**
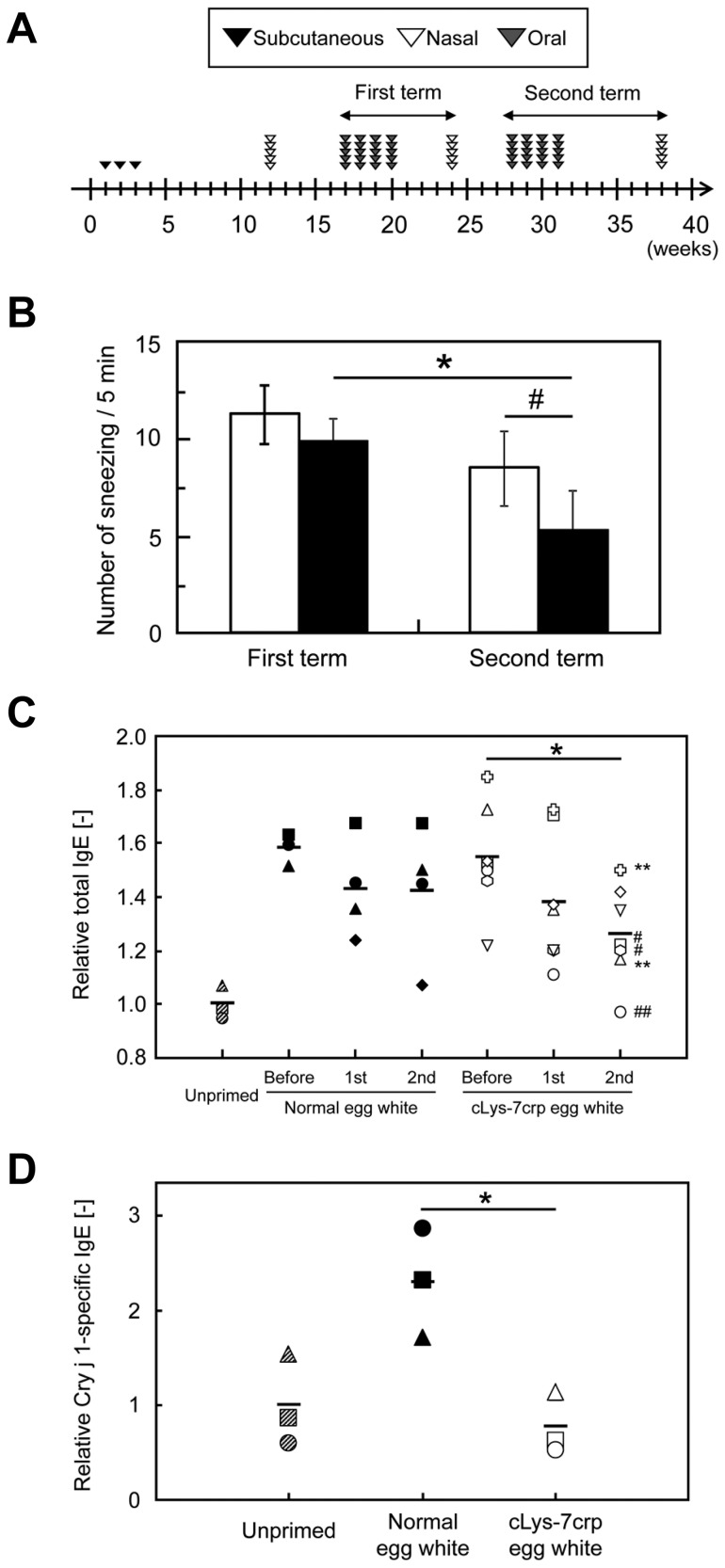
Therapeutic efficacy of cLys-7crp-containing egg white through oral administration. (**A**) Administration schedule. Cedar pollen extract was subcutaneously injected into mice three times. A mouse model of allergic rhinitis specific for Cj pollenosis was generated by intranasal challenge with cedar pollen extracts for five days per week. After five weeks, egg white containing cLys-7crp produced by GM chickens (#23) or normal egg white (0.3 ml per day each) was administered orally to Cj allergy mice five days per week. Finally, antigen challenge using cedar pollen extracts were performed. Oral administration of egg white and allergen challenges were repeated twice. (**B**) The number of sneezes measured after egg white therapy. Immediately after the final intranasal administration of Cj allergen for each therapeutic trial, the number of sneezes in mice fed with cLys-7crp-containing (closed columns) or normal (open columns) egg white were counted. *p<0.05, significantly different between the first and second therapeutic trial term of orally-administered cLys-7crp-containing egg white produced by GM chickens. ^#^ *p*<0.05, significantly different between normal and cLys-7crp-containing egg white treated mice. (**C–D**) Amounts of total IgE (**C**) and Cry j 1-specific IgE (**D**) in the serum of mice fed with normal or cLys-7crp-containing egg white after intranasal challenge with cedar pollen extracts. “Before”, “1st” and “2nd” represent serum samples collected from Cj pollen-sensitized mice before oral administration, after the first therapeutic trial and after the second therapeutic trial, respectively. Each symbol represents an individual mouse. Slanting-striped symbols represent the unprimed mice group; closed symbols represent the group fed with normal egg white; open symbols represent the group fed with cLys-7crp-containing egg white. *p (mean) <0.05, ^#^ *p*<0.05 (open hexagonal shape and square), **p<0.01 (open cross shape and triangle) and ^##^ *p*<0.005 (open circle); significantly different compared with levels before administration of cLys-7crp-containing egg white produced by GM chickens.

A murine Cj pollinosis model was developed to evaluate the therapeutic efficacy of oral administration of cLys-7crp produced in the egg white of a GM hen. We used B10.S mice, because the mouse strain is a good murine model for the tentative preclinical study of patients with Cj pollinosis [30], [33]. The administration plan of Cj allergen and cLys-7crp egg white is shown in Fig. 4A. Nine weeks after sensitization by three serial subcutaneous injections of cedar pollen extract together with adjuvant, the Cj allergen was administrated intranasally for 5 days. Immediately after the final intranasal administration of Cj allergen, the number of sneezes was counted for 5 min. Sneezing number has been employed as one of evaluation criteria for murine models of allergic rhinitis [13], [14], [34], [35]. The mean number of sneezes for sensitized mice was 15.3±4.2, while the value for unprimed mice (control) was 1.5±1.3. This observation was consistent with a previous report [31], indicating that the Cj pollinosis model mice could be successfully generated.

To investigate whether oral administration of cLys-7crp-containing egg white would have efficacy in a mouse model of Cj pollinosis, egg white derived from a GM hen or normal egg white derived from a non-GM hen as the control, were orally administered to Cj pollinosis-induced mice. Following the intranasal challenge of Cj allergen after a series of oral administration of egg white (the first term of oral administration), the number of sneezes was slightly decreased for the mice treated with cLys-7crp-containing egg white compared with controls. Following intranasal challenge after a second round of treatment, the number of sneezes was further decreased for the mice fed with cLys-7crp-containing egg white compared with the controls fed with normal egg white (Fig. 4B).

Since the oral administration of cLys-7crp-containing egg white exhibited a therapeutic effect in Cj pollinosis mice, we measured the total IgE content in the serum of each mouse before and after the administration of cLys-7crp-containing or normal egg white (Fig. 4C). The total IgE levels for three of four mice administered with normal egg white were unchanged, although one mouse showed a decrease in total IgE levels (closed diamond). However, there was a significant reduction in total IgE in five out of seven mice (71.4%) treated with cLys-7crp-containing egg white, and in one mouse the total IgE levels (open circle) decreased similar to naïve mice. Moreover, Cry j 1-specific IgE levels in the serum after the second term of oral administration were measured (Fig. 4D). Cry j 1-specific IgE levels were reduced in the same cLys-7crp-fed mice that showed a significantly reduced total IgE (open circles, open triangles and open squares in Fig. 4C).

**Figure 5:**
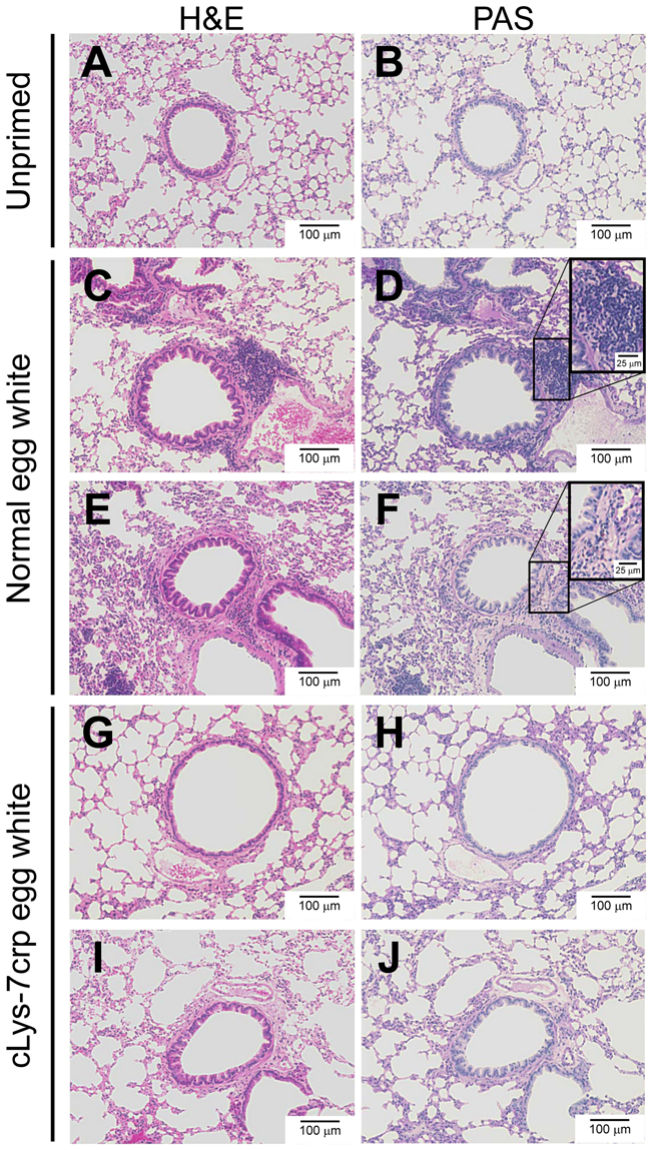
Histological observation for evaluation of allergen-induced inflammatory cell infiltration in lungs of treated mice. Lung samples from unprimed mice (**A, B**) and Cj pollinosis model mice fed with normal egg white (**C–F**) or cLys-7crp-containing egg white (**G–J**) were harvested 24 h after the intranasal challenge, fixed with paraformaldehyde and stained with H&E (**A, C, E, G, I**) or PAS (**B, D, F, H, J**). (**C–J**) The sections were prepared from the lung samples of mice presented with the symbols of closed diamonds (**C, D**), closed circles (**E, F**), open cross shapes (**G, H**) and open diamonds (**I, J**) in Fig. 4.

We examined cellular infiltration by inflammatory response in lungs of the treated mice. Lung tissues harvested from the mice were applied for histological analyses. To assess inflammatory cells such as eosinophils, H&E staining sections of the lung tissues were observed (Fig. 5A, 5C, 5E, 5G, 5I). No inflammatory cells were observed in the lung of control mice (Fig. 5A), while the lung tissue sections from Cj pollinosis mice fed with normal egg white showed obvious peribronchial inflammatory infiltrates (Fig. 5C, 5E). The pathological feature was significantly diminished in the lung specimens from Cj pollinosis mice fed with cLys-7crp-containing egg white (Fig. 5G, 5I). The lung tissue sections were also evaluated for mucus production in the airway epithelium by PAS staining (Fig. 5B, 5D, 5F, 5H, 5J). Although some PAS-positive cells were observed in the peribronchial region of cellular infiltration, notable mucus production in bronchial lumens was not observed in the lung tissues for the mice fed with normal egg white (Fig. 5D, 5F). On the other hand, no PAS-positive cells were detected for the mice fed with cLys-7crp-containing egg white (Fig. 5H, 5J). Thus, cellular infiltration in lungs of the Cj pollinosis model mice fed with cLys-7crp-containing egg white was significantly reduced compared with the mice fed with normal egg white. These results suggested that oral administration of cLys-7crp-containing egg white might be an effective method to induce mucosal tolerance to the Cj allergen.

## Discussion

We generated GM chickens expressing T-cell epitope peptides for the Cj allergen as a fused protein with cLys. The direct oral feeding of egg white containing T-cell epitopes for Cj pollinosis could inhibit the symptoms of pollen allergy in Cj pollinosis model mice. To date, many recombinant proteins such as antibodies [24], [36] and cytokines [25], [26], [37] have been produced by GM chickens. Generally, such therapeutic proteins should be purified prior to their use. In this study, we attempted to use the eggs containing the therapeutic proteins produced by GM chickens as a therapeutic food. To generate 7crp-producing GM chickens, we first constructed a retroviral vector plasmid expressing 7crp. However, retroviral vector production was impaired using the plasmid. Therefore, the production format of 7crp was changed to a fusion protein with cLys, which allowed the production of high titers of retroviral solution. The GM chickens generated using the retroviral vector stably produced cLys-7crp in egg white for over a year (data not shown), but the production level was not high (1.3±0.2 μg/ml) compared with our previous reports [24], [26]. cLys-7crp was not produced in the serum and other organs of GM chickens although RNA transcripts were detected in various tissues and organs of the GM chicken. T-cell epitopes are usually composed from hydrophobic amino acids that fit the peptide-binding groove of an MHC molecule. 7crp was identified as a hydrophobic protein having the hydrophobicity/hydrophilicity value of –0.22 (hydrophobic) as determined by the method of Hopp and Woods [38]. Thus, cLys-7crp might be sequestered at the cell membrane during the secretion pathway through the endoplasmic reticulum/Golgi, and might be processed through the degradation pathway in the cells. However, in oviduct cells, cLys-7crp may be secreted because of the highly alkaline condition of egg white. However, the detailed mechanism requires further elucidation. cLys-7crp production may be improved by changing the production format, including the epitope order and fusion partner.

There are several routes for administration of allergens for immunotherapy, such as subcutaneous, intralymphatic, transcutaneous, sublingual and oral administrations. Among them, the side effects of sublingual and oral hyposensitization therapy are generally mild, and the procedure is simple. Thus, the sublingual and oral administration of allergens has been considered more beneficial for treatment of Cj pollinosis patients [17]. Moreover, T-cell epitope peptides of allergens have been used for hyposensitization immunotherapy against allergenic diseases such as cedar pollen [12], cat [39] and peanut [40] allergies. Thus, induction of oral tolerance by peptide immunotherapy may be a useful strategy for radical treatment and/or the prevention of allergic diseases if gut immunity can be effectively activated, and with a low risk of side effects [13], [14] when compared with using whole allergen. In recent years, the mechanisms of oral tolerance through the gut have been gradually elucidated [41]–[43]. After ingested antigen and food proteins are absorbed through microfold cells and intestinal epithelial cells in gut-associated lymphoid tissue, mucosal microenvironmental factors such as transforming growth factor-β and interleukin-10 promote the differentiation of adaptive regulatory T-cells that subsequently induce antigen-specific suppressive responses. Various antigen-presenting intestinal dendritic cells are also required to induce the adequate differentiation of T cells in Peyer's patches and mesenteric lymph nodes by delivering immunosuppressive signals [15]. Thus, the complex network between immune cells results in the formation of an antigen-specific immunosuppressive microenvironment in the gut, leading to the induction of oral tolerance to target proteins. In this study, direct oral feeding of cLys-7crp-containing egg white to Cj pollinosis mice might induce suppressive immune responses against the cedar pollen allergens establishing oral tolerance.

B10.S mice were used to generate the Cj pollinosis mouse model, because major T-cell epitopes identified in Cry j 1-sensitized B10.S mice coincided with the most prevalent epitopes in human patients [30]. Oral administration of cLys-7crp-containing egg white to Cj pollinosis mice caused the down-regulation of total and specific IgE in the serum although the difference of total IgE amounts between unprimed and allergy model mice was not as high compared with a previous report [4]. The total IgE levels were not significantly decreased in two mice treated with cLys-7crp-containing egg white (open diamond and open inverted triangle in Fig. 4C). For these two mice, the seven dominant T-cell epitopes selected in this study might not completely induce immunological tolerance, because the cedar pollen extract used to sensitize the mice contains other antigens apart from Cry j 1 and Cry j 2. Nevertheless, these results suggest that the cedar pollen allergy symptoms of Cj pollinosis model mice were moderated by the oral feeding of cLys-7crp-containing egg white produced by GM chickens.

In the present study, although oral administration of cLys-7crp to the Cj pollinosis mice induced immunotolerance based on hyposensitization, the cLys-7crp dose and feeding plan (frequency and interval, 0.3 μg of cLys-7crp fed to mice for 5 days a week, repeated for 4 weeks in one term) were not optimized. Since the cLys-7crp production level in egg white was not high, the maximum single dose was limited by the volume (0.3 ml) of egg white injectable to a mouse using the feeding needle. In the study using 7crp-containing transgenic rice, 560 μg of 7crp was fed daily to Cj pollinosis mice for 32 days to induce oral immunotolerance [14]. Thus, the daily dose of cLys-7crp-containing egg white was approximately 1,800-fold less than that of the previous study. Since chicken egg white proteins such as lysozyme and ovalbumin are relatively immunogenic, the chicken lysozyme region of cLys-7crp and egg white itself may act as an adjuvant. Interestingly, a higher efficiency of oral tolerance was induced when transgenic rice containing a fusion protein of cholera toxin B subunit with allergen-specific T-cell epitopes was orally administered to mice, compared with the administration of transgenic rice containing only allergen-specific T-cell epitopes [44]. Thus, to achieve a stronger induction of immunotolerance, an elevated dose of cLys-7crp and/or a change in the feeding plan may be a feasible approach. However, to increase a single dose treatment, it would be necessary to enhance the expression level of cLys-7crp in egg white or to concentrate cLys-7crp by rough purification. Therapeutic effects on moderation of the allergic symptoms for the oral administration of cLys-7crp-containing egg white to Cj pollinosis model mice were observed for other sets of therapeutic experiments, in which feeding schedule of cLys-7crp-containing egg white was changed (data not shown).

In conclusion, we generated GM chickens producing cLys-7crp in egg white. When the egg white containing cLys-7crp was orally administered to Cj pollinosis-sensitized mice, sneezing was suppressed upon allergen challenge concurrent with a significant reduction of total and Cry j 1-specific IgE levels in the serum of mice. Of particular note, specific IgE levels in the serum were decreased to that of unprimed mice. These results indicate that eggs derived from GM chickens can be used as edible pharmaceuticals.
